# Ghrelin *O*-acyltransferase (GOAT) is expressed in prostate cancer tissues and cell lines and expression is differentially regulated *in vitro* by ghrelin

**DOI:** 10.1186/1477-7827-11-70

**Published:** 2013-07-23

**Authors:** Inge Seim, Penny L Jeffery, Laura de Amorim, Carina M Walpole, Jenny Fung, Eliza J Whiteside, Rohan Lourie, Adrian C Herington, Lisa K Chopin

**Affiliations:** 1 Ghrelin Research Group, Translational Research Institute - Institute of Health and Biomedical Innovation, Queensland University of Technology, 37 Kent St, Woolloongabba, Queensland, 4102, Australia; 2 Australian Prostate Cancer Research Centre, Queensland, Princess Alexandra Hospital, 199 Ipswich Road, Brisbane, Queensland, 4102, Australia; 3 Mater Medical Research Institute, Mater Health Services, University of Queensland, South Brisbane, Queensland,, 4103, Australia; 4 Department of Pathology, Mater Health Services, South Brisbane, Queensland, 4103, Australia

**Keywords:** Acyl-modification, Ghrelin, GOAT, Prohormone convertase, Prostate cancer

## Abstract

**Background:**

Ghrelin is a 28 amino acid peptide hormone that is expressed in the stomach and a range of peripheral tissues, where it frequently acts as an autocrine/paracrine growth factor. Ghrelin is modified by a unique acylation required for it to activate its cognate receptor, the growth hormone secretagogue receptor (GHSR), which mediates many of the actions of ghrelin. Recently, the enzyme responsible for adding the fatty acid residue (octanoyl/acyl group) to the third amino acid of ghrelin, GOAT (ghrelin *O*-acyltransferase), was identified.

**Methods:**

We used cell culture, quantitative real-time reverse transcription (RT)-PCR and immunohistochemistry to demonstrate the expression of GOAT in prostate cancer cell lines and tissues from patients. Real-time RT-PCR was used to demonstrate the expression of prohormone convertase (PC)1/3, PC2 and furin in prostate cancer cell lines. Prostate-derived cell lines were treated with ghrelin and desacyl ghrelin and the effect on GOAT expression was measured using quantitative RT-PCR.

**Results:**

We have demonstrated that GOAT mRNA and protein are expressed in the normal prostate and human prostate cancer tissue samples. The RWPE-1 and RWPE-2 normal prostate-derived cell lines and the LNCaP, DU145, and PC3 prostate cancer cell lines express GOAT and at least one other enzyme that is necessary to produce mature, acylated ghrelin from proghrelin (PC1/3, PC2 or furin). Finally, ghrelin, but not desacyl ghrelin (unacylated ghrelin), can directly regulate the expression of GOAT in the RWPE-1 normal prostate derived cell line and the PC3 prostate cancer cell line. Ghrelin treatment (100nM) for 6 hours significantly decreased GOAT mRNA expression two-fold (P < 0.05) in the PC3 prostate cancer cell line, however, ghrelin did not regulate GOAT expression in the DU145 and LNCaP prostate cancer cell lines.

**Conclusions:**

This study demonstrates that GOAT is expressed in prostate cancer specimens and cell lines. Ghrelin regulates GOAT expression, however, this is likely to be cell-type specific. The expression of GOAT in prostate cancer supports the hypothesis that the ghrelin axis has autocrine/paracrine roles. We propose that the RWPE-1 prostate cell line and the PC3 prostate cancer cell line may be useful for investigating GOAT regulation and function.

## Background

Ghrelin is a 28 amino acid peptide, which is post-translationally cleaved by furin-like proteases from a larger (117 amino acid) preproghrelin protein [1,2]. Following cleavage of the signal peptide, proghrelin can be post-translationally octanoylated by the enzyme ghrelin *O*-acyltransferase (GOAT) at the third residue of mature ghrelin, a serine, to form acylated ghrelin [3,4]. Proghrelin is cleaved by one of a number of enzymes, including prohormone convertase (PC) 1/3, PC2 or furin [1,5] to produce the 28 amino acid mature ghrelin peptide. Ghrelin acylation is required for it to bind and activate the classical ghrelin receptor, the growth hormone secretagogue receptor (GHSR1a) [2,6]. A non-octanoylated form of ghrelin, desacyl ghrelin, (or unacylated ghrelin), circulates in the blood at higher levels than octanoylated ghrelin [7,8], but does not activate the GHSR1a. Desacyl ghrelin and ghrelin have functions that are mediated by an alternative ghrelin receptor that has not yet been identified [9]. The acylated form of ghrelin (termed ghrelin) is a multifunctional peptide hormone that plays a role in a range of cellular processes, including the regulation of growth hormone and insulin release, appetite, gut motility, and metabolism and it has roles in the immune, cardiovascular and reproductive systems [10].

Evidence is also emerging that ghrelin plays a role in regulating cancer progression [11,12]. Ghrelin and GHSR1a expression has been reported in a number of cancers [12] including breast [13,14], prostate [15-20], testicular [21], and endometrial cancer [22,23]. We have previously demonstrated that ghrelin and GHSR1a are expressed in prostate cancer and prostate cancer cell lines [16,18]. Prostate cancer tissues and cell lines express a novel ghrelin mRNA isoform, that encodes the 28 amino acid hormone, and this isoform is regulated by insulin [19]. Prostate cancer cell lines secrete the ghrelin peptide and exogenous ghrelin treatment stimulates cell proliferation in prostate cancer cell lines and this is mediated through the ERK1/2 MAPK pathway [16,18]. It has recently been demonstrated that a fluorescein-labelled ghrelin (1–18) probe which binds prostate cancer tissue can be used to distinguish between benign prostatic disease and prostate cancer, and therefore, may be a useful diagnostic tool [20].

The recently discovered ghrelin acylation enzyme, ghrelin *O*-acyltransferase (GOAT), encoded by the gene *MBOAT4* (membrane bound *O*-acyl transferase 4) [3,4], is emerging as a promising diagnostic and therapeutic target for regulating appetite and conditions related to the ghrelin axis, such as obesity, insulin resistance and type 2 diabetes mellitus [24]. GOAT, like ghrelin, is produced by the stomach, and also in a range of peripheral tissues including the normal human prostate [25] and in breast cancer [26]. The expression of the GOAT gene (*MBOAT4*) is regulated by growth factors and hormones, including leptin [27,28], somatostatin [28] and ghrelin itself, but not by desacyl ghrelin [28].

In this report, we extend our previous studies, demonstrating the expression and function of the ghrelin axis in prostate cancer [16,18], to demonstrate that prostate-derived tissues and cell lines express the necessary enzymes required to produce the 28 amino acid acylated ghrelin, which may play a role in prostate cancer proliferation [16,18]. Moreover, we examined the ability of exogenous ghrelin and desacyl ghrelin to alter the expression of GOAT mRNA in prostate-derived cell lines.

## Methods

### Cell culture

Cell lines were obtained from the American Type Culture Collection (ATCC, Rockville, MD). The non-tumourigenic RWPE-1 (ATCC CRL-11609) and the transformed, tumourigenic RWPE-2 (ATCC CRL-11610) prostate epithelium-derived cell lines were cultured in Keratinocyte Serum Free Medium (KSFM) (Invitrogen, Carlsbad, CA) supplemented with 50 μg/mL bovine pituitary extract and 5 ng/mL epidermal growth factor (Invitrogen). The PC3 (ATCC CRL-1435), DU145 (ATCC HTB-81), and LNCaP (ATCC CRL-1740) prostate cancer cell lines were maintained in Roswell Park Memorial Institute 1640 medium (RPMI 1640, Invitrogen) with 10% New Zealand Cosmic Calf Serum (HyClone, South Logan, UT, USA) supplemented with 100 U/mL penicillin G and 100 μg/mL streptomycin (Invitrogen).

All cell lines were passaged at two to three day intervals on reaching 70% confluency using either 0.25% Trypsin/EDTA (Invitrogen) or stable Trypsin-like Enzyme (TrypLE Express) (Invitrogen). Cell morphology and viability was monitored by microscopic observation and regular *Mycoplasma* testing was performed by PCR (Universal Mycoplasma Detection Kit, ATCC). All general disposable cell culture laboratory ware was from Nagle Nunc International (Roskilde, Denmark).

### Ghrelin and desacyl ghrelin treatments

RWPE-1, LNCaP, DU145, and PC3 prostate cancer cell lines were grown in flasks and serum-starved overnight when they reached 70% confluency. Cells were treated with ghrelin (0, 100 and 1000nM) or desacyl ghrelin (0, 100 and 1000nM) (Mimotopes, Melbourne, Australia) in phenol red-free RPMI1640 media (Invitrogen), in serum free conditions for 0, 1 and 6 hours at 37°C in a humidified atmosphere containing 5% CO_2_. Cells were washed twice in phosphate buffered saline and removed from flasks using cell scrapers, and cell pellets were stored at −80°C for RNA extraction.

### RNA extraction

Total RNA was harvested from cultured cells using QIAshredder and RNeasy Plus Mini kits (QIAGEN, Hilden, Germany) according to the manufacturer’s instructions and stored at −80°C. RNA concentration and purity were measured using a NanoDrop ND-1000 spectrophotometer (Thermo Fisher Scientific, Waltham, MA). Contaminating genomic DNA was removed by *DNase I* digestion (amplification grade, Invitrogen), and 1.0 μg total RNA was reverse transcribed using the SuperArray RT^2^ First Strand Kit (SABiosciences, Frederick, MD), or using SuperScript III First strand synthesis system (Invitrogen).

### Quantification of GOAT, furin, prohormone convertase (PC) 1/3 and PC2 mRNA expression in prostate tissues and cell lines using real-time RT-PCR

The expression of GOAT, PC1/3, PC2 and furin mRNA was investigated in prostate cancer cell lines. Quantitative real-time RT-PCRs were performed using the AB 7500 sequence detection system (Applied Biosystems, AB, Foster City, CA) in a total reaction volume of 25 μl using 2 × SYBR Green PCR Master Mix (AB). Primers for GOAT/*MBOAT4* (cat. no PPH63501A), furin (*FURIN* PPH09618A), PC1/3 (*PCSK1* PPH10136A), and PC2 (*PCSK2* PPH16826E) were purchased from SABiosciences. Expression was normalised to *18S* ribosomal RNA (forward 5′-TTCGGAACTGAGGCCATGAT-3′ and reverse 5′-CGAACCTCCGACTTTCGTTCT-3′). Fold changes were quantified as 2^-(ΔCt sample-ΔCt control)^, as described previously [29]. Statistical analysis was performed using the Student’s *t*-test, with duplicate data from two independent experiments, or one-way analysis of variance (ANOVA) with Tukey’s *post-hoc* analysis for multiple group analysis, with P < 0.05 considered to be statistically significant.

### Quantification of GOAT mRNA expression in prostate tissues using real-time RT-PCR

Real-time RT-PCR was used to determine GOAT mRNA expression levels in samples from tissues of normal prostate and prostate cancer patients using OriGene TissueScan qPCR Prostate Cancer cDNA panels (panel II, OriGene, Rockville, MD). cDNA panels were derived from eight normal tissues and a range of tumour tissues. Quantitative real-time RT-PCR was performed using GOAT (*MBOAT4*) primers as described above. Expression levels were normalised to β-actin (using primers supplied with the cDNA panel). For cDNA panels, each gene was evaluated on separate, identical array plates, which were loaded with equal amounts of cDNA per well, as described by the manufacturer.

### Immunohistochemistry using tissues and prostate cancer cell lines

A prostate cancer tissue microarray (TMA) was obtained from the Cooperative Prostate Cancer Tissue Resource (TMA 2 Gleason) with approval from the QUT Human Research Ethics Committee. The array contains prostate cancer tissue cores obtained from radical prostatectomy specimens from 250 patients, with Gleason scores of 4 (3 cases), 5 (31 cases), 6 (53 cases), Gleason 7 (54 cases), 8 (52 cases), 9 (54 cases) and Gleason 10 (2 cases). The array also included 58 high-grade prostate intraepithelial neoplasia (HGPIN) cases, 18 cases of benign prostatic hyperplasia (BPH), 14 non-diseased donor prostates, as well as the LNCaP, DU145 and PC3 prostate cancer cell lines.

Immunohistochemical staining was performed using a polyclonal antibody to human *MBOAT4*/GOAT (raised in goat) using formalin-fixed, paraffin-embedded human prostate cancer tissue. Sections were incubated with anti-MBOAT4/GOAT antibody (1:100) (ab99449, Abcam, Cambridge, UK) overnight at 4°C followed by incubation with goat HRP-polymer-linked secondary antibody-based detection (GHP516L, BioCare Medical, Concord, CA) according to the manufacturer’s instructions. Human stomach was used as a positive control. This antibody was affinity purified by the manufacturer using the peptide to which it was raised and its specificity was tested by pre-absorbing the antibody with GOAT peptide.

### Bioinformatics

Using the Human Protein Atlas, the tissue localisation of PC1/3, PC2 and furin expression was explored in prostate tissues [30].

## Results and discussion

### GOAT mRNA is expressed in human prostate and prostate cancer tissue and in prostate cell lines

**Figure 1:**
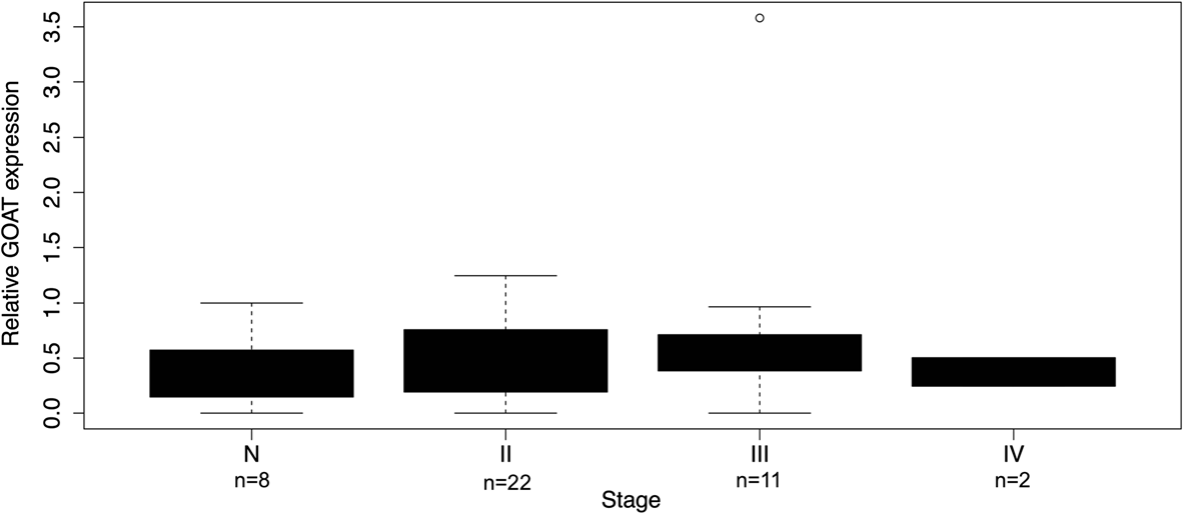
**Boxplot showing relative levels of GOAT/*MBOAT4 *mRNA expression.** Expression in normal prostate tissue (denoted N) and prostate cancer tissue (stages II to IV) performed using an OriGene TissueScan qPCR Prostate Cancer panel. Data were normalised to β-actin and are represented as fold changes relative to expression of transcripts in a normal prostate sample (1.0). GOAT was not differentially expressed in any group (Kruskal-Wallis test; over all *p* = 0.56).

To determine if human prostate cancer tissues and cell lines express the enzyme which acylates ghrelin, GOAT (*MBOAT4*), we performed quantitative real-time RT-PCR. GOAT was expressed in most normal and prostate cancer tissues, but was not expressed at higher levels in prostate cancer tissue compared to normal prostate tissue specimens, regardless of cancer stage (Figure 1), or grade (data not shown). Our findings that GOAT was not overexpressed in prostate cancer specimens compared to benign prostatic tissue contrasts recent findings in breast cancer, where it has been reported that GOAT mRNA was overexpressed in 40 high grade tumors (G3) compared to four normal breast samples [29].

**Figure 2:**
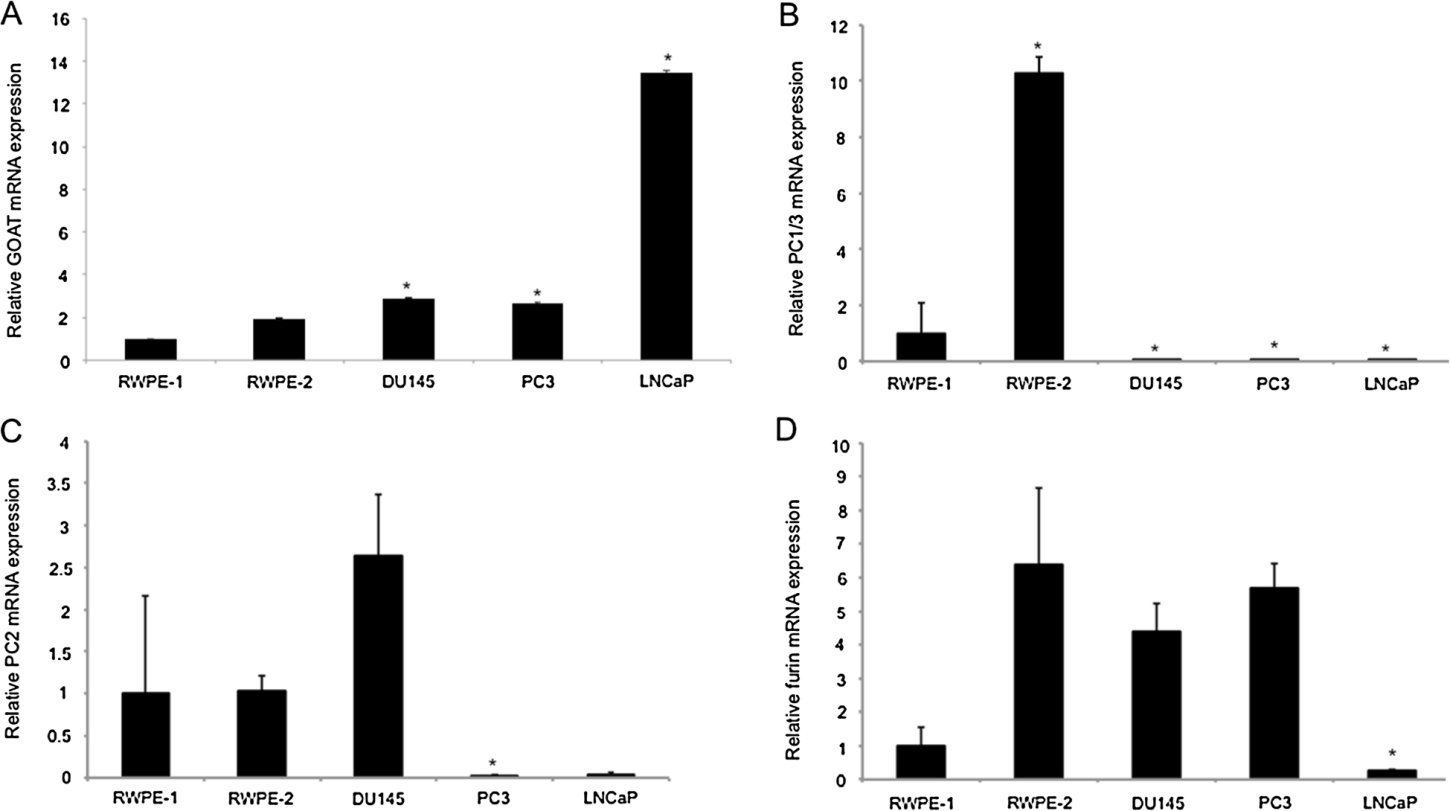
**Real-time quantitative RT-PCR assays.** Assays were performed to determine the relative mRNA expression levels of **(A)** GOAT (*MBOAT4*), **(B)** PC1/3 (*PCSK1*), **(C)** PC2 (*PCSK2*), and **(D)** furin (*FURIN*) in the RWPE-1 and RWPE-2 normal-prostate derived cell lines and the DU145, LNCaP and PC3 prostate cancer-derived cell lines. Data are expressed relative to the RWPE-1 cell line mRNA levels (set as 1) and are presented as means ± S.E.M. (n = 2, assays performed in duplicate) and compared by one-way ANOVA, followed by Tukey’s *post-hoc* analysis.* = *P* < 0.05 versus the RWPE-1 cell line.

Having confirmed that GOAT/*MBOAT4* mRNA is expressed in prostate cancer, we examined its expression in normal prostate-derived and prostate cancer-derived cell lines. GOAT mRNA expression was significantly higher in the LNCaP (13.4-fold), DU145 (2.9-fold) and PC3 (2.6-fold) prostate cancer cell lines compared to the normal prostate-derived RWPE-1 cell line (P < 0.05) (Figure 2A). GOAT expression in the tumourigenic, prostate epithelial-derived cell line, RWPE-2 (which is derived from RWPE-1 cells subjected to K-ras oncogene transfection) [31], was not significantly different from the RWPE-1 cell line (Figure 2).

### Prohormone convertase expression in prostate-derived cell lines

To determine if prostate cancer cell lines have the enzymatic machinery necessary for preproghrelin processing, we screened a range of prostate cancer cell lines using quantitative real-time RT-PCR for the protease processing enzymes prohormone convertase (PC)1/3, PC2 and furin, which can each process proghrelin to the 28 amino acid ghrelin peptide in a mutually exclusive manner [1]. All of the prostate cell lines tested expressed at least one of the processing enzymes necessary to produce the mature ghrelin peptide (Figure 2B-C). This correlates with our previous finding that immunoreactive ghrelin is present in the conditioned media from PC3 and LNCaP prostate cancer cell lines [16]. The LNCaP cell line expressed relatively low levels of furin, PC1/3 and PC2 expression, however (Figure 2B-C). PC1/3 expression in the DU145, PC3 and LNCaP prostate cancer cell lines was significantly lower than the RWPE-1 normal prostate derived cell line (P < 0.05) (Figure 2B) and similarly, no PC1/3 expression was demonstrated in the LNCaP and DU145 cell lines in a previous study [32]. PC1/3 expression was approximately 11-fold higher in the RWPE-2 cell line compared to the RWPE-1 cell line, from which this cell line was derived by transformation of the RWPE-1 cell line with v-Ki-ras [31] (Figure 2B). Increased PC1/3 expression has been associated with androgen withdrawal and neuroendocrine differentiation in a PC310 prostate cancer cell line mouse xenograft model [33]. PC2 was expressed in the RWPE-1, RWPE-2, and DU145 cell lines and at lower levels in the PC3 and LNCaP cell lines (Figure 2C). Although we have demonstrated PC2 expression in the DU145 cell line, expression of the PC2 transcript was reported to be absent in this cell line in a previous study [32]. PC1/3 is moderately to strongly expressed in the cytoplasm of 90% of prostate cancer samples (n = 18) in the Human Protein Atlas [30]. Furin expression has previously been demonstrated in the LNCaP and DU145 cell lines, [32] and furin mRNA was expressed in all of the cell lines tested in the current study. Furin expression in the LNCaP cell line was significantly lower than the RWPE-1 (the reference) cell line (Figure 2D).

Prohormone convertase (PC) expression has been associated with a number of cancers, including gynaecological cancers, and lung, skin and gastric cancer [34-36] and PCs could provide novel targets for cancer treatments. The prohormone convertase, PACE4, has been linked with increased prostate cancer cell proliferation and cancer progression, and a peptide that targets PACE4 and furin inhibits prostate cancer proliferation [32,37]. PACE4 has not been demonstrated to have a role in processing preproghrelin, however.

Recently, we identified a novel insulin-regulated ghrelin mRNA isoform that is expressed in prostate cancer cell lines and in prostate cancer [38]. As prostate cancer cell lines produce ghrelin, and exogenous ghrelin treatment stimulates cell proliferation in prostate cancer cell lines, we hypothesized that the ghrelin axis may play an autocrine/paracrine role in prostate cancer [39]. Our previous studies demonstrated that ghrelin and the ghrelin receptor, GHSR, are expressed at the mRNA and protein levels in prostate cancer cell lines and prostate cancer specimens [16,38,39]. A study demonstrating that a fluorescein-labelled ghrelin (1–18) probe binds prostate cancer tissue with high specificity also demonstrated GHSR1a expression in the PC3 and LNCaP prostate cancer cell lines and the BPH-1 cell line [20]. The finding that prostate cancer cells and tissues also express GOAT, the enzyme required for ghrelin octanoylation, and the prohormone convertases that are required for ghrelin processing, provides further support for this hypothesis [16,18].

### Immunohistochemical staining of human prostate tissues and cell lines

**Figure 3:**
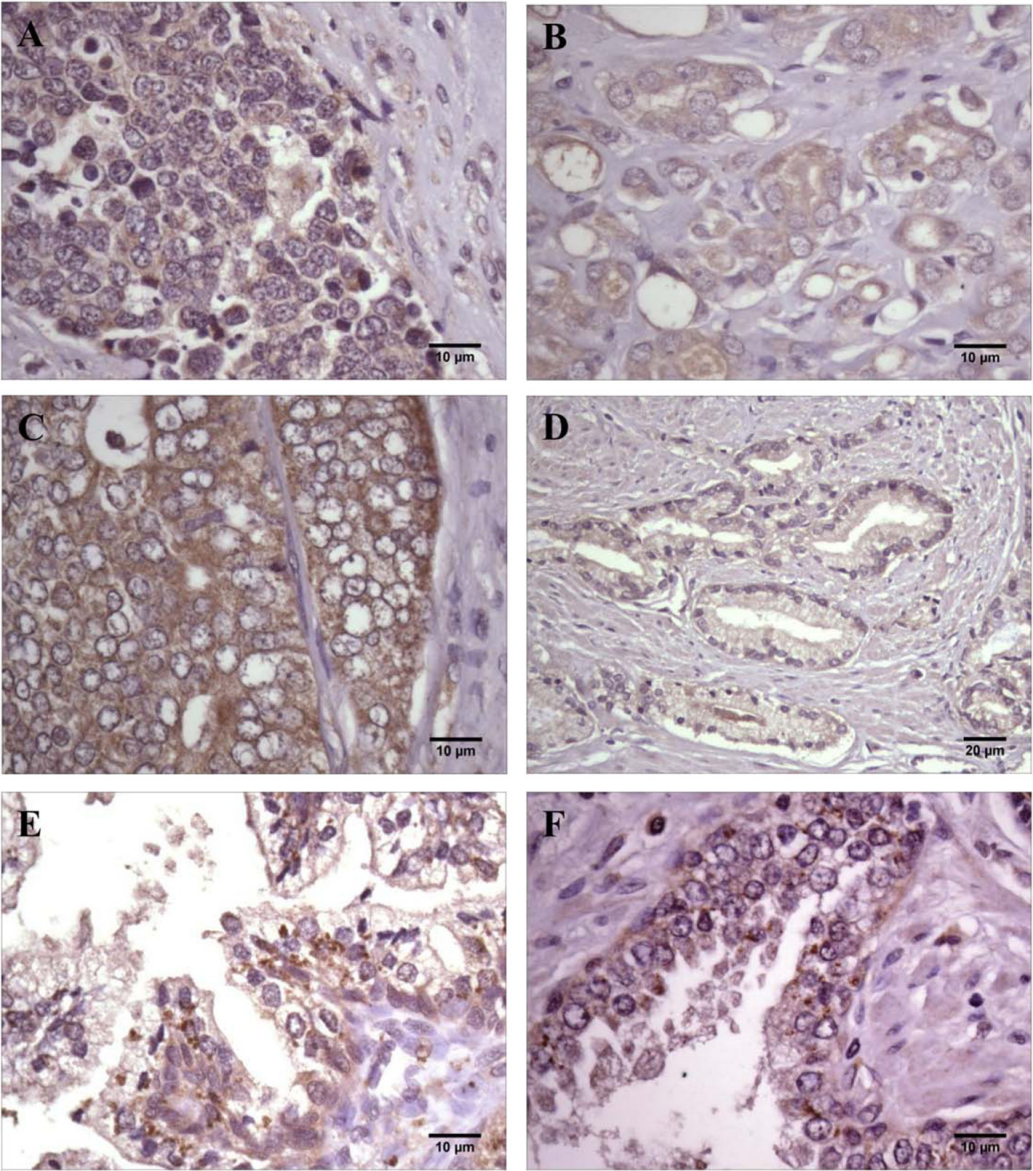
**Representative photomicrographs of ghrelin *O*-acyltransferase (GOAT) immunoreactivity in prostate cancer specimens and in benign prostatic hyperplasia (BPH).** Diffuse cytoplasmic staining was detected in **(A)** poorly differentiated cancer specimens, **(B)** Gleason grade 4 + 5 and **(C)** Gleason grade 4+ 2. **(D)** No immunoreactivity was detected in some cancers (Gleason grade 3 +3) demonstrating the heterogeneity of GOAT expression in prostate cancer. More granular dot-like staining was present in **(E**, **F)** BPH sections. Scale bars are as indicated on micrographs.

We next demonstrated that GOAT protein is expressed in prostate cancer tissues and benign prostatic hyperplasia (BPH) samples using immunohistochemistry (Figure 3). GOAT immunoreactivity in prostate cancer is characterized by heterogenous cytoplasmic staining predominantly in prostate epithelial cells (Figure 3). Some cancers lacked GOAT expression, however (Figure 3D). As observed using qRT-PCR (at the mRNA level), immunohistochemical screening of 250 prostate tissue specimens demonstrated that GOAT protein is not differentially expressed in human prostate cancer. There was no correlation between GOAT expression and tumour grade and GOAT was not differentially expressed in benign prostatic hyperplasia compared to prostate cancer (Figure 3A, B and C). Immunohistochemical staining of BPH specimens demonstrated light cytoplasmic staining with some membrane accentuation. Many of the hyperplastic cells were noted to have sharply stained cytoplasmic granules giving a granular, dot-like immunoreactivity (Figure 3E, F). Cytoplasmic staining for GOAT was also present in LNCaP, DU-145 and PC3 prostate cancer cell lines (data not shown).

### Ghrelin peptide hormone treatment regulates GOAT expression in cultured prostate-derived cells

**Figure 4:**
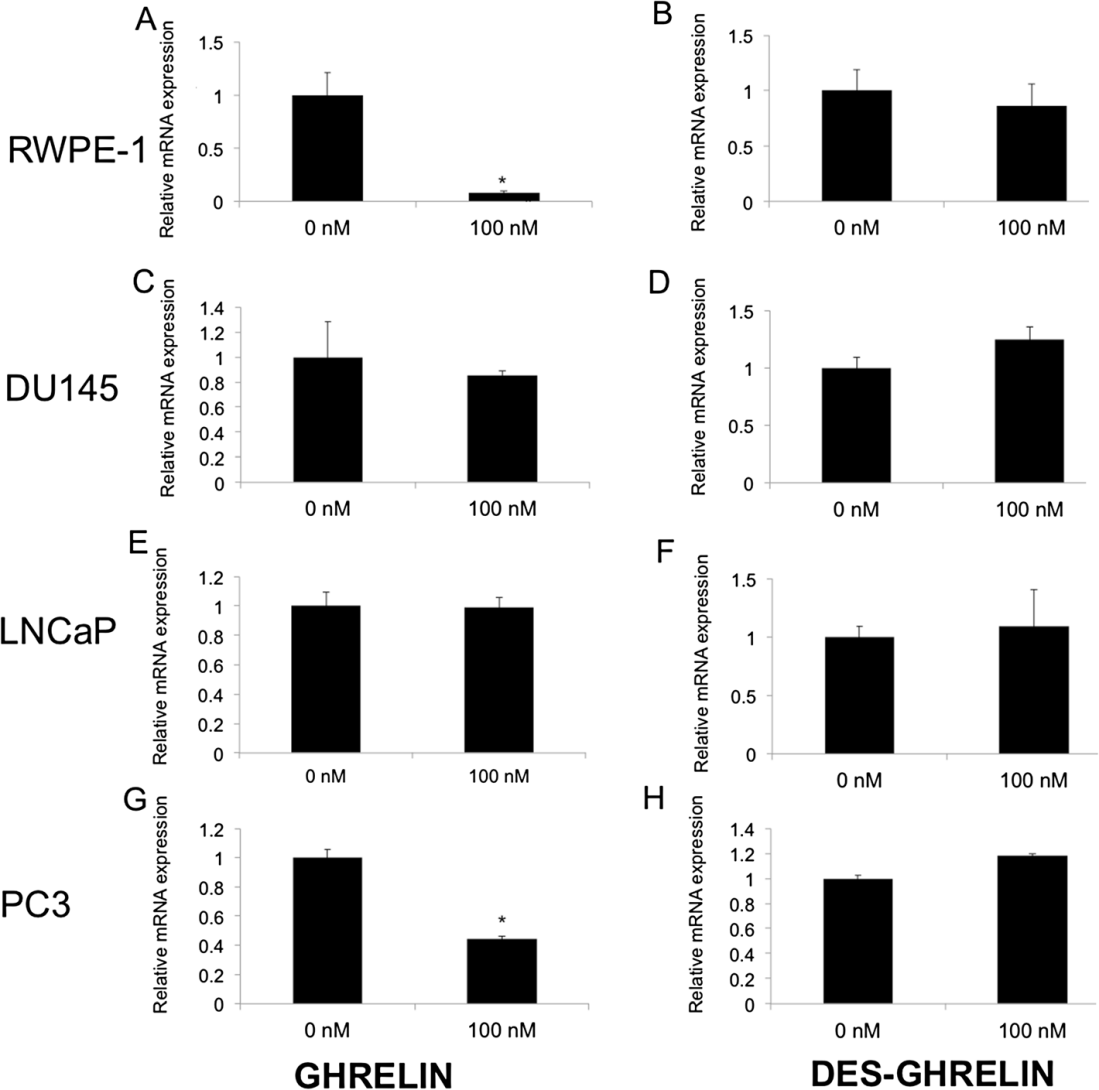
**Real-time quantitative RT-PCR analysis of GOAT gene expression in response to ghrelin and desacyl ghrelin treatments for 6 hours.** GOAT mRNA expression in ghrelin treated **(A)** RWPE-1 normal prostate derived cell line, and the **(C)** DU145, **(E)** LNCaP and **(G)** PC3 prostate cancer cell lines and desacyl ghrelin-treated **(B)** RWPE-1, **(D)** DU145 and **(F)** LNCaP and **(H)** PC3 cell lines. Data is represented as means and standard error of two technical replicates from two independent replicate experiments (n = 2). * *P* < 0.05 indicates values that differ significantly (Student’s *t*-test) from vehicle-treated control (set at 1).

Finally, we examined whether desacyl ghrelin or acylated ghrelin regulate GOAT mRNA expression in prostate cancer cell lines. Treatment with 100nM (Figure 4A, G), or 1000nM (data not shown) acylated ghrelin for 6 hours caused a statistically significant (*P* < 0.05) decrease in the expression of GOAT mRNA in the RWPE-1 normal prostate derived and the PC3 prostate cancer cell lines compared to untreated controls. No changes in GOAT mRNA expression were observed 1 hour after ghrelin or desacyl ghrelin treatment in this cell line (data not shown). Treatment with acylated ghrelin had no effect on GOAT mRNA expression in the DU145 and LNCaP prostate cancer cell lines, however (Figure 4C, E). Desacyl ghrelin treatment did not alter the expression of GOAT/*MBOAT4* mRNA in the RWPE-1, DU145, LNCaP or PC3 cell lines (Figure 4B, D, F, H).

By downregulating GOAT expression, ghrelin would downregulate its octanoylation, altering the balance between acylated ghrelin and desacyl ghrelin in the prostate. As acylated ghrelin can bind and activate the GHSR1a (the active form of the ghrelin receptor), but desacyl ghrelin does not activate GHSR1a, GOAT could act as a switch in the prostate. Desacyl ghrelin and ghrelin can have different effects in different tissues, and changing the balance between these two forms of ghrelin in the prostate may also alter the physiological response. Although desacyl ghrelin is functional in a number of systems, but does not bind the classical ghrelin receptor, GHSR1a, it is likely to function through the widely hypothesised alternative ghrelin receptor [12].

GOAT modifies the ability of ghrelin to stimulate appetite and it is required for behaviours associated with hedonic feeding [40]. Ghrelin and GOAT have also been shown to play a role in protecting against hypoglycaemia with severe caloric restriction [41,42], however, this is currently highly controversial and has been refuted [43]. GOAT inhibitors show promise for the development of therapeutics for improving glucose tolerance and preventing weight gain [44]. These inhibitors could be useful in prostate cancer patients and may reduce autocrine/paracrine ghrelin-stimulated cell proliferation. GOAT inhibitors might also be useful for improving glucose tolerance in patients with hyperinsulinaemia and impaired glucose tolerance which results from androgen withdrawal therapy for advanced prostate cancer [45].

There have been few studies into the regulation of *MBOAT4*, the gene encoding GOAT, however, it is clear that it is under tight endocrine control. In contrast to our findings in prostate cancer cells, ghrelin treatment upregulates GOAT expression (2-fold) in primary mouse pituitary cells [28]. Ghrelin has no effect on GOAT mRNA expression in cultured murine and human chondrocytes, however [46]. Conversely, based on forced over-expression studies in mice, it has been proposed that GOAT may regulate the ghrelin gene at the post-transcriptional level *in vivo,* although the precise mechanisms have yet to be determined [47]. This intriguing biological regulatory circuit warrants further studies.

Evidence is also emerging that a number of hormones that control the growth hormone (GH) axis, or energy metabolism, regulate GOAT mRNA expression. Growth hormone releasing hormone (GHRH) upregulates GOAT mRNA levels, while somatostatin (which inhibits GH release) down-regulates GOAT [28]. The peptide hormone leptin (which like ghrelin plays a key role in regulating energy intake and expenditure, including appetite) increases GOAT mRNA levels *in vitro* and *in vivo*[27,28]. Insulin down-regulates GOAT expression in the INS-1 rat insulinoma cell line [48], but does not affect expression of GOAT in primary mouse pituitary cells [28]. A recent study demonstrated that testosterone increases GOAT mRNA levels in rat gastric tissue [49]. It would be interesting to examine whether GOAT is regulated by androgens in cultured prostate cancer cells in future studies.

Although ghrelin regulated GOAT expression in the PC3 prostate cancer cell line, it did not influence GOAT expression in the LNCaP or DU145 prostate cancer cell lines. This is surprising, as the PC3, DU145 and LNCaP cell lines express ghrelin and GHSR1a and ghrelin treatment stimulates cell proliferation in these cell lines [16,18]. The different response may be due to the fact that the three prostate cancer cell lines treated with ghrelin are of different metastatic origins [50]. The PC3 prostate cancer cell line is derived from a bone metastasis, the DU145 cell line is derived from a brain metastasis and the LNCaP prostate cancer cell line is derived from a lymph node metastasis. The PC3 and DU145 cell lines are androgen-independent, while the LNCaP cell line is androgen dependent. The differential response to ghrelin treatment may reflect the different metastatic origins of these cells, and their gene signatures may have been influenced by their *in vivo* microenvironments. Ghrelin also downregulated expression of GOAT mRNA in the RWPE-1 normal prostate derived cell line, however. Although this cell line is derived from normal prostate epithelium and is non-tumourigenic, it is derived from cells which have been transfected with human papilloma virus 18, and this may affect their behavior [31]. Conversely, the regulation of GOAT by acyl ghrelin may be a feature of normal prostate physiology which has been lost in the LNCaP and DU145 cell lines, but not in the PC3 cell line. In future studies, it would be useful to dissect the differences between ghrelin and desacyl ghrelin induced gene expression in the PC3 and RWPE-1 cell lines by transcriptomic or proteomic analyses. Finally, no reliable quantitative assays for GOAT protein levels have been reported [24]. The development of such assays will greatly aid in probing the interplay between ghrelin, desacyl ghrelin and GOAT.

## Conclusions

This is the first study to demonstrate the expression of GOAT/*MBOAT4* mRNA in prostate cancer tissues and cell lines, and to examine the effect of ghrelin on GOAT expression in cultured prostate cancer cell lines. We found that GOAT mRNA and protein is expressed at similar levels in the normal prostate and prostate cancer tissues (independent of their disease stage or grade). Normal prostate and prostate-cancer derived cell lines expressed mRNA encoding the enzymes, PC1/3, PC2 or furin, which are necessary to cleave ghrelin from the proghrelin precursor, and GOAT, the enzyme that octanoylates ghrelin. This study provides further evidence that ghrelin may play an autocrine/paracrine role in prostate cancer. Finally, we have found that ghrelin, but not desacyl ghrelin, can directly down-regulate the expression of GOAT in the RWPE-1 normal derived cell line and the PC3 prostate cancer cell line, but not in the DU145 and LNCaP prostate cancer cell lines. This suggests that the regulation of GOAT expression may be cell-type specific. We propose that the RWPE-1 prostate cell line and the PC3 prostate cancer cell line may be useful model cell lines to investigate GOAT/*MBOAT4* regulation and function.

## Abbreviations

ERK1/2: Extracellular-signal related kinase 1/2; GH: Growth hormone; GHSR: Growth hormone secretagogue receptor; GOAT: Ghrelin *O*-acyltransferase; MAPK: Mitogen activated protein kinase; MBOAT: Membrane bound *O*-acyltransferase; PC: Prohormone convertase; RT-PCR: Reverse transcription polymerase chain reaction; TMA: Tissue microarray.

## Competing interests

The authors declare that they have no competing interests.

## Authors’ contributions

IS, CW, JF and PJ performed RNA extractions, real-time RT-PCR and data analysis. PJ performed the immunohistochemical assays and with RL analysed and interpreted the data. LA and CW performed cell culture experiments. IS, PJ, LA, EW, AH, CW, JF and LC played roles in the design of the project, the interpretation of the data and writing and editing the manuscript. All authors read and approved the final manuscript.
